# Feasibility of IG and TCR rearrangements quantification in ctDNA for monitoring clinical response in pediatric lymphomas

**DOI:** 10.3389/fgene.2026.1875204

**Published:** 2026-06-25

**Authors:** Milena R. Marusco, Rudolph R. Vera, Caroline de O. Lopes, Natacha A. Migita, Dieila G. De Lima, Felipe L. T. Silva, Guilherme N. N. Giusti, João Meidanis, Camila M. M. Daiggi, Camila Z. Mouco, José A. Yunes, Mariana C. Maschietto, Patricia Y. Jotta

**Affiliations:** 1 Research Center, Boldrini Children’s Center, Campinas, Brazil; 2 Department of Genetics, Institute of Biological Sciences, Federal University of Amazonas (UFAM), Manaus, Brazil; 3 Institute of Computing, State University of Campinas (UNICAMP), Campinas, Brazil; 4 Oncology, Boldrini Children’s Center, Campinas, Brazil; 5 Department of Translational Medicine, State University of Campinas, Campinas, Brazil; 6 Postgraduate Program in Genetics and Molecular Biology, Institute of Biology, State University of Campinas (UNICAMP), Campinas, Brazil

**Keywords:** ctDNA (circulating tumor DNA), ig/TCR gene rearrangements, liquid biopsy, minimal residual disease (MRD), pediatric lymphoma

## Abstract

**Introduction:**

Lymphomas are malignancies of the lymphatic system characterized by clonal rearrangements of immunoglobulin (Ig) and T-cell receptor (TCR) genes. While diagnosis relies on tissue biopsy, circulating tumor DNA (ctDNA) analysis offers a minimally invasive approach for real-time disease monitoring. This study evaluated next-generation sequencing (NGS) for the detection and quantification of Ig and TCR rearrangements in ctDNA from pediatric lymphoma patients.

**Methods:**

Forty cases (21 Hodgkin and 19 non-Hodgkin lymphomas) were analyzed using NGS with BIOMED-2 and EuroClonality primers.

**Results:**

Clonal rearrangements were identified in tumor DNA from 23 patients, yielding 54 rearrangements. For 19 patients, paired tumor (gDNA) and plasma (cfDNA) at diagnosis samples were analyzed. Most of the rearrangements identified in these tumor samples (n = 48) were found in the corresponding diagnostic cfDNA (n = 43, 89.6%). Patients who relapsed showed significantly higher cfDNA levels at diagnosis, and in one case, ctDNA increase preceded clinical or radiological relapse.

**Discussion:**

These findings support liquid biopsy as a feasible tool for monitoring disease dynamics and early relapse detection in pediatric lymphoma.

## Graphical Abstract

**Figure:**
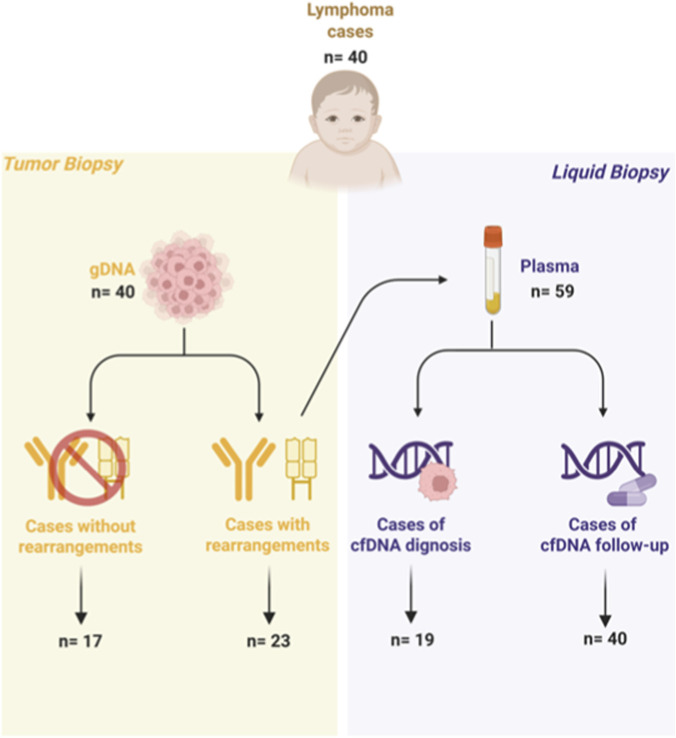
Flowchart of sample analysis at the time of diagnosis, with a focus on identifying clonal markers for plasma samples (cfDNA) used in liquid biopsy. Created in BioRender. Jotta, P (2026) https://BioRender.com/h7uxen4.

## Introduction

Lymphoma encompasses a heterogeneous group of diseases that accounts for approximately 10%–15% of childhood cancers and are broadly classified into two main categories: Hodgkin lymphoma (HL) and non-Hodgkin lymphoma (NHL), based on morphological, immunophenotypic, and genetic features. The long-term survival rate of pediatric lymphoma exceeds 80%, varying according to the subtype and the stage (Minard-Colin et al., 2015). The diagnostic gold standard is the tissue biopsy, which enables subtype classification according to histopathological and molecular characteristics (Alaggio et al., 2022). However, tissue biopsy provides a partial representation of the disease, without fully accounting for the spatial and temporal heterogeneity (Gerlinger et al., 2012). Tumor response to treatment is conventionally assessed by imaging methods that may lack the sensitivity to detect minimal residual disease (MRD) (Sachpekidis et al., 2025).

The analysis of circulating tumor DNA (ctDNA) enables the detection of tumor-derived material in peripheral blood in a less invasive nature and allows the capture of the intratumoral heterogeneity as well as the monitoring of disease response to treatment (Huang et al., 2024; Bartolomucci et al., 2025). Cancer patients have higher levels of circulating DNA (0–1000 ng/mL) compared to healthy individuals or patients with other pathologies, with the ctDNA representing up to 1% of the total DNA circulating in the bloodstream (Diaz and Bardelli, 2014). Because both normal and tumoral cells contribute to circulating free DNA (Schwarzenbach et al., 2011), the amount of total cfDNA is not *per se* a sensitive marker for the monitoring of tumor progression. Mutations (Werner et al., 2025; Desch et al., 2019; Diehl et al., 2007), copy number alterations (Silva et al., 2023; Ruas et al., 2023), DNA methylation (Kristensen et al., 2016; Tian et al., 2023; Ding et al., 2017) and microsatellite instability can be detected in the ctDNA (Pirosa et al., 2025). The ctDNA is considered a dynamic biomarker of tumor biology because it rapidly degrades in the bloodstream and thus, the ctDNA level changes may help to monitor cancer response to treatment providing a non-invasive, real-time assessment of tumor dynamics (Ossandon et al., 2018; Heitzer et al., 2015).

For patients with lymphoma, the ctDNA was used for monitoring tumor burden and allowed the early detection of relapse, providing complementary information to imaging modalities, such as positron emission tomography/computed tomography (PET/CT). In aggressive lymphoma subtypes, including diffuse large B-cell lymphoma (DLBCL) and HL, the ctDNA was associated with treatment response, minimal residual disease (MRD), and allowed detection of tumor relapse before conventional radiological techniques (Bartolomucci et al., 2025; Roschewski et al., 2016; Roschewski et al., 2015a; Camus et al., 2020; Roschewski et al., 2022; Rossi et al., 2019).

Immunoglobulin (Ig) and T-cell receptor (TCR) gene rearrangements are widely used as molecular targets to detect minimal residual disease (MRD) in pediatric acute lymphoblastic leukemia (ALL), providing valuable diagnostic and prognostic information regarding relapse risk (Huang et al., 2017; Assumpção et al., 2011; van Dongen et al., 2003; Langerak et al., 2012). Given that lymphomas and leukemias originate from lymphoid cells, the characterization of Ig and TCR rearrangements may also serve as basis for liquid biopsy approaches in patients with lymphomas (van Krieken et al., 2007). This strategy may allow disease monitoring when tissue samples are limited and contribute to a more comprehensive analysis of tumor heterogeneity.

## Methods

### Patient selection and sample collection

This study included consecutive patients diagnosed with any type of lymphoma who were admitted between March 2020 and June 2025 at Boldrini Children’s Center, provided biological samples were available for the analyses. Eligible patients had both tumor and plasma samples available at the institutional biobank, with informed consent for research. This study was approved by the local Research Ethics Committee (CAAE: 44219021.6.0000.5376).

Tumor samples were obtained from tumor biopsies stored in RNAlater solution (Invitrogen). CtDNA was obtained from total blood collected in EDTA tubes and processed in up to 24 h.

Using PET/CT, the radiological standard method to define “remission” (complete response) and relapse/progression in lymphoma was the five-point Deauville Scale: complete remission is defined as scores 1–2 which means absence of ^18^F-FDG uptake or minimal uptake (lower than the mediastinum and liver) represents a complete metabolic response to treatment. Relapse or progression in lymphoma is defined by scores 4–5: moderate to high FDG uptake (higher than the mediastinum/liver) that represents persistent disease or disease progression, and score 3: indeterminate uptake (requires additional clinical evaluation). The Deauville criteria are integrated with the Lugano Classification (2014), which standardizes: Complete Response (CR): Disappearance of all evidence of disease; Partial Response (PR): ≥50% reduction in tumor burden; Stable Disease (SD): <50% change in lesion size and Progressive Disease: ≥50% increase in tumor burden or appearance of new lesions.

### Sample preparation and DNA extraction

Tumor DNA was extracted, using GenELUTE Mammalian Genomic DNA Miniprep kit (SIGMA-Aldrich, St. Louis, MO, United States of America; cat. ID: G1N70-1 KT). DNA was quantified using the Qubit dsDNA HS Assay Kit (Invitrogen™, Waltham, MA, United States of America; cat. ID: Q32853). DNA integrity was assessed by electrophoresis on a 0.8% agarose gel.

CfDNA was extracted using the QIAmp MinElute ccfDNA kit (Ref. 28904265 - Cytiva) and eluted twice in 15 µL of the elution solution. Between 1 mL and 1.9 mL (average of 1.5 mL) of plasma was collected from each patient. Samples were quantified using the Qubit dsDNA HS Assay Kit (Ref. Q32854 - Invitrogen). Approximately 1 ng of cfDNA was analyzed on the 2,100 Bioanalyzer (Agilent).

### Identification of DNA rearrangements in the tumor tissue and cfDNA samples

NGS libraries were prepared using a two-step nested PCR previously described (Giusti et al., 2023) to identify Ig and TCR rearrangements using primers published by the BIOMED-2 (van Dongen et al., 2003) (IgH, TRD, IgK and IgL) and by EuroClonality (TRG) (Brüggemann et al., 2019) consortiums. For tumor samples, 50 ng DNA was submitted for the first PCR. The clonal rearrangements are defined using a frequency threshold exceeding 5% in tumor samples (Giusti et al., 2023; Kurtz et al., 2015). For plasma diagnostic and follow-up samples, 25 ng and 2 ng of cfDNA, respectively, were subjected to the first PCR, which employed primers for the rearrangements previously identified in the corresponding tumor sample. Also, due to the small size of cfDNA, the IgH Biomed two framework region 3 (FR3) primer was used instead of framework region 2 (FR2), because it generates smaller amplicons (van Dongen et al., 2003).

To determine the minimum amount of cfDNA, the limit of detection (LOD) was calculated using a cfDNA positive for an IgH rearrangement diluted in a negative cfDNA sample, with concentration starting at 2 ng in a 1:1 dilution. The analysis of the 11 dilution points showed that the method provided reliable results in samples with 0.03125 ng of positive cfDNA, which was therefore defined as the minimum amount of cfDNA reliable for the experiments (Supplementary Material Figure S1).

Ig and TCR rearrangements in linear plasmids were added to the first PCR reaction as a spike in controls (Giusti et al., 2023). 5 μL of the first PCR reaction were used in a second PCR to add the adapters and index (Nextera XT Index Kit - Illumina), followed by cleanup with Agencourt Ampure XP Beads (Beckman Coulter). The library was resuspended in 10 mM Tris-Cl buffer, pH 8.5 (elution buffer (EB) - Qiagen) and quantified using the Qubit dsDNA HS Assay Kit. The library concentration was corrected by the size of the fragments, according to the Bioanalyzer results. Tumor samples were sequenced on MiSeq (Illumina) using MiSeq Reagent Kit v3 (150-cycle) (MS-102-3001 - Illumina), generating 100,000 reads per gDNA sample. CfDNA samples were sequenced on NextSeq 550 equipment (Illumina) with reagents from the NextSeq 500/550 v2.5 Kits (150-cycle) (20024904 - Illumina). For each cfDNA sample, one million reads were generated per rearrangement identified in the diagnostic tumor. Sequencing data were uploaded to the BaseSpace Sequence Hub v7.38.0 (Illumina) for sample demultiplexing and to generate FASTQ files.

### Analysis of the NGS results using the VIDJIL software

FASTQ files were analyzed on the High-Throughput Analysis of V(D)J Immune Repertoire (VIDJIL) platform (Duez et al., 2016; Giraud et al., 2014; Villarese et al., 2022; Langlois de Septenville et al., 2022). Rearrangements identified in the tumor samples at diagnosis were searched in the cfDNA from plasma at diagnosis and follow-up. Considering that NGS quantifies the number of reads and not directly the number of cfDNA molecules, spike-ins were used to construct an internal standard curve, allowing the number of reads to be converted into an absolute number of molecular copies, and thus, applied as a measure of the amount of cfDNA, similarly to the minimal residual disease quantification used for acute lymphoblastic leukemia (Giusti et al., 2023). To assess rearrangement productivity, each clonotype was analyzed using VIDJIL according to its predicted reading frame status. This analytical step allowed us to characterize the productivity profile of each rearrangement type, following the standard criteria in which productive rearrangements correspond to sequences that preserve an in-frame V(D)J reading frame.

### Associations between Ig/TCR data and patients’ characteristics

Analyses were performed using GraphPad Prism, version 9.0 (GraphPad Software). Differences in cfDNA or ctDNA levels between groups were assessed using Mann–Whitney test or Kruskal–Walli’s test, according to the number of groups being compared. Correlations between ctDNA and tumor characteristics were evaluated by applying the Spearman’s one-tailed test.

## Results

### Identification of rearrangements in pediatric lymphomas

This study included 40 pediatric patients diagnosed with lymphoma classified according to the World Health Organization (WHO) 2017 blue book (Borowitz et al., 2008), comprising HL (n = 21, 52.5%, being 20 nodular sclerosis (NSHL) and one nodular lymphocyte-predominant (NLPHL) subtypes) and 19 NHL, divided in 14 B-cell lymphomas: 11 (27.5%) Burkitt lymphoma (BL), one lymphoblastic lymphoma (LBL-B), and two diffuse large B-cell lymphomas (DLBCL); and 5 T-cell lymphomas: two anaplastic large cell lymphoma (ALCL) and three lymphoblastic lymphoma (LBL-T). Rearrangements of Ig (IgH, IgK and IgL) and TCR genes (TRG and TRD) were screened in all tumor samples, according to the groups (Supplementary Material Figure S2). Rearrangements of Ig (IgH, IgK and IgL) and TCR genes (TRG and TRD) were screened in all tumor samples. The frequency of each rearrangement in lymphoma groups is presented in Supplementary Figures S2C,D, and in B-cell lymphoma in Supplementary Figures S1F, S2E. The Supplementary Material Figure S3 summarizes key clinical aspects of the pediatric patients included in the study, including vital status (A), Ann Arbor staging classification (B), age at diagnosis (C), and the distribution of cases with metastasis (D) or relapse (E).

Ig and TCR rearrangements were identified in the tumor of 57.5% (n = 23) of the patients, with the frequency of gene rearrangements varying among the types of lymphomas. The subtypes with the highest proportion of rearrangements detected were LBL-B (100%, n = 1/1), followed by BL (90.9%, n = 10/11) and LBL-T (66.7%, n = 2/3). DLBCL showed rearrangements in 50% (n = 1/2) of cases, while NSHL showed rearrangements in 45% (n = 9/20). NLPHL (0%, n = 0/1) and ALCL (0%, n = 0/2) did not show detectable rearrangements in the samples evaluated (Table 1; Supplementary Material Figure S2A).

**TABLE 1:** Identification of Ig/TCR rearrangements in pediatric lymphoma tumor samples.

*Tumo n = 40*	*Positive patients*	*Detected rearrangements*
NSHL n = 20	n = 9 (45%)	IgH - n = 5 (25%) IgK - n = 6 (30%) IgL - n = 6 (30%) TRD - n = 1 (5%)
NLPHL n = 1	None	None
DLBCL n = 2	n = 1 (50%)	IgH - n = 1 (50%) IgK - n = 1 (50%) IgL - n = 1 (50%)
BL n = 11	n = 10 (90,9%)	IgH - n = 10 (90.9%) IgK - n = 7 (63.6%) IgL - n = 5 (45.4%)
LBL-B n = 1	n = 1 (100%)	IgH - n = 1 (100%) TRD - n = 1 (100%)
ALCL n = 2	None	None
LBL-T n = 3	n = 2 (66,6%)	TRG - n = 2 (66.6%) TRD - n = 1 (33.3%)

A total of 54 rearrangements were identified, IgH (n = 18, 32.7%), IgK (n = 15, 27.3%), IgL (n = 14, 25.5%), TRG (n = 4, 7.3%) and TRD (n = 3, 5.6%) in 23 of the 40 (57.5%) tumor samples, with 18 samples presenting more than one rearrangement, as exemplified by patient 14, that presented six rearrangements (2 IgK, 1 IgH, 1 IgL and 2 TRD), while five samples had only one rearrangement type (Table 1; Supplementary Material Figure S1B).

Among the 54 rearrangements identified in the 23 tumors, 44 (81.5%) were productive and 10 (18.5%) were out of frame. The highest productivity rate was observed in IgH rearrangements, with 94.4% (n = 17/18), followed by IgL with 85.7% (n = 12/14), IgK with 73.3% (n = 11/15). Among T-cell receptors, TRD presented 66.7% (n = 2/3) productive rearrangements and TRG 50.0% (n = 2/4).

### Characterization of cfDNA in pediatric patients diagnosed with different types of lymphomas

From the 40 patients, 59 samples of plasma were available: 19 samples collected at diagnosis and 40 samples collected during follow-up (**Graphical abstract)**. The yield of cfDNA from 19 diagnostic samples was highly variable with no differences in cfDNA concentration observed across lymphoma subtypes. From the 20 L H, 8 (40%) relapsed and 12 (60%) was stage IV, from which 6 had a diagnostic sample with an rearrangement identified, resulting in 3 (50%) that relapsed and 2 (33%) stage IV. From the 11 B L, 1 (9%) relapsed and 3 (27%) was stage IV, from which 10 had a diagnostic sample with an rearrangement identified, resulting in 1 (10%) that relapsed and 3 (30%) stage IV. The TCL were grouped with the BCL forming the NHL group (Supplementary Material Figure S2) that was used for comparison in the cfDNA analysis (Supplementary Material Figure S4I). For both groups, plasma concentration of cfDNA (ng/mL) at diagnosis or follow up did not associate with metastasis (Supplementary Figures S4C–F) while there was a tendency toward a correlation between cfDNA concentration and tumor stage, with stage IV patients exhibiting the highest cfDNA levels (Supplementary Figures S4A,B). NHL patients who relapsed had higher cfDNA plasma concentrations at diagnosis compared to those who did not relapse, although this analysis is biased due to the small number of samples (*p = 0.0256) (Supplementary 4G–4H**)**.

**FIGURE 1:**
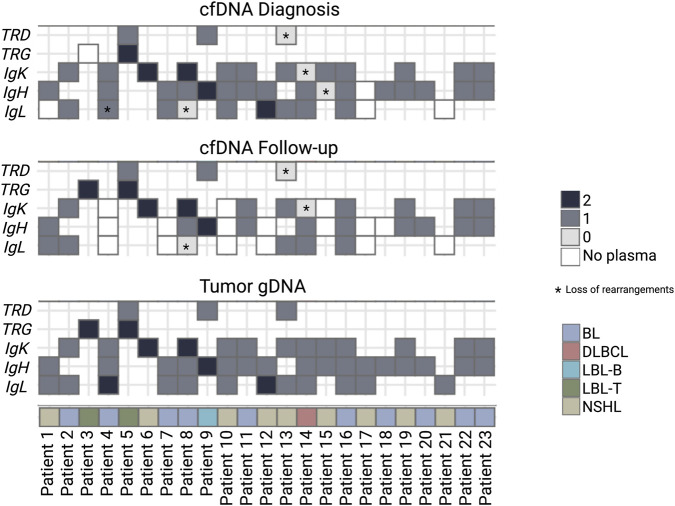
Ig/TCR rearrangements detected in tumor DNA (gDNA), cfDNA in the diagnosis and follow-up of patients with lymphoma.

For 19 patients, paired tumor (gDNA) and plasma (cfDNA) at diagnosis samples were analyzed. Most of the rearrangements identified in these tumor samples (n = 48) were found in the corresponding diagnostic cfDNA (n = 43, 89.6%). In patients #13, #10 and #14, all with three rearrangements found in the tumor sample, one of these was not detected in diagnostic cfDNA. Although five rearrangements were detected only in gDNA: 2 IgL, 1 IgH, 1 IgK and 1 TCRD, all patients had at least one rearrangement found at diagnosis cfDNA (Figure 1). The Supplementary Material Figure S5 shows which rearrangements of Ig and TCR detected in tumor DNA (gDNA) were also detected in cfDNA in the diagnosis and follow-up of patients with lymphoma.

For the 19 patients who had rearrangements identified in the diagnostic plasma sample, 15 had available follow-up cfDNA samples, two patients did not have diagnostic cfDNA, but had follow up samples. Thirty out of 54 rearrangements were found at some point of the follow-up plasma samples (Figure 1).

**FIGURE 2:**
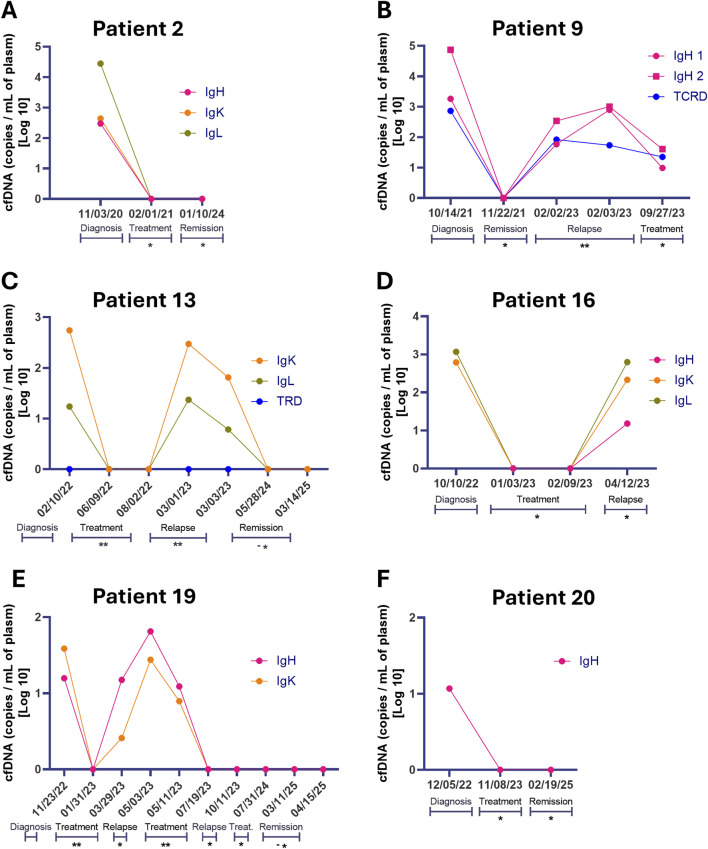
Monitoring of patients through cfDNA (copies/mL of plasma) using Ig and TR rearrangements. The x-axis shows sampling dates and clinical stages, and the y-axis represents ctDNA copies/mL of plasma for clonal Ig/TCR rearrangements detected in plasma. Each panel corresponds to one patient. In (*) indicates the timing of imaging evaluations on the x-axis, whereas (**−)** indicates that imaging evaluation was not performed. **(A)** Patient 2: Detection of Ig/TCR DNA disapeared following end of treatment. Detection of Ig/TCR decreased during treatment and increased during relapse for patients **(B)** 9, **(C)** 13, and **(D)**. 16, **(E)** Patient 19: Ig/TCR ctDNA levels increased before the first but before the second relapse, **(F)** Patient 20: Detection of Ig/TCR DNA disapeared following end of treatment.

For seven patients (#2 – Figure 2, #5, #8, #14, #20, #22, #23 – Supplementary Material Figure S6), the rearrangement levels disappeared with the treatment and remission. For three patients (#9, #13, #16 – Supplementary Material Figure S6) the ctDNA levels decreased with the treatment and increased when relapse occurred. For patient #6 (Supplementary Material Figure S6), the only sample available after diagnosis was the relapse, which was detected by ctDNA, but was lower than the diagnostic levels. The ctDNA levels showed the clinical response of treatment for these patients (Figure 1; Supplementary Material Figure S6). Patient #3 (Supplementary Material Figure S6) only had relapse samples, the ctDNA levels increased after 2 months and the patient died in 15 days after the last point of evaluated plasma. The details of the treatment in each point of the follow-up collected sample was shown in Supplementary Material Table S1.

For patient #19 (Figure 1), an increase of the Ig/TCR ctDNA levels preceded the clinical diagnosis of the relapse by CT, but a second relapse was not predicted by the Ig/TCR ctDNA levels. Tumor sample from the second relapse was not available to investigate whether the Ig/TCR was lost along tumor progression.

## Discussion

Monitoring tumor progression using ctDNA is a promising tool for patients with cancer, especially when tumor tissue is limited or unfeasible over time (Pascual et al., 2022; Diaz and Bardelli, 2014). This non-invasive approach is particularly advantageous for cancer patients, especially in the pediatric setting, as it reduces the need for recurrent invasive procedures, preserving their quality of life without compromising the rigor of clinical follow-up.

This study aimed to evaluate the use of ctDNA as a dynamic biomarker for pediatric patients diagnosed with lymphomas, especially because it overcomes limitations of traditional methods such as imaging tests or serial biopsies (van Krieken et al., 2007; Scherer et al., 2016). Moreover, reinforcing the potential of ctDNA for patients with lymphoma, an adult prospective study suggested that cfDNA could be used to define early response during the DLBCL treatment (Bohers et al., 2018).

One study obtained clonal sequences using IgH primers in 23.8% of HL with a multiplex BIOMED two consortium primer set (Chute et al., 2008), with no differences regarding IgH (n = 5, 23.8%) e IgK (n = 5, 23.8%). The different frequencies of IgH (15.5%) and of IgK (20.7%) clonal rearrangements have been reported in HL (Tapia et al., 2012). The frequency threshold of 5% may have limited the rearrangements in the tumor, as Reed-Sternberg cells represent 0.1%–2% in HL (Küppers et al., 2012), which can explain the low frequency of rearrangements found. This is a limitation of the software used for analysis that was optimized for major clones in leukemia (Duez et al., 2016; Giraud et al., 2014; Villarese et al., 2022; Langlois de Septenville et al., 2022). Nevertheless, once identified in the tumor, that rearrangement was specifically targeted in the cfDNA.

As for B-cell lymphomas, IgH and IgK clonal rearrangements were detected in 47 patients with DLBCL, with IgK being detected more frequently than IgH (74% and 53% respectively), probably due to the higher rate of somatic hypermutation (Roschewski et al., 2015b; Sebastián et al., 2012; Alcoceba et al., 2024). Our study had just one patient with IgH, IgK and IgL rearrangements and 85% of patients (n = 17) with an IgH rearrangement, although all B-Lymphomas with a rearrangement had IgH. The IgK was present in only 61.5% (n = 13) of our cases. Previously, detection of IGK gene rearrangements was reported in 100% of B lymphoma samples by NGS, while IGHV-IGHD-IGHJ and IGHD-IGHJ rearrangements were observed in 71% and 93% of cases, respectively (Scheijen et al., 2019). This discrepancy may be explained by the fact that our cohort comprised only pediatric patients. Considering that these data show a predominance of productive rearrangements, their frequency suggests that Ig rearrangements could be an interesting target for monitoring the treatment of pediatric lymphomas.

Although the BIOMED-2 consortium primers are widely validated for Ig/TCR clonality assessment, amplicon-based approaches present important limitations in germinal center-derived lymphomas, particularly BL and HL, which frequently harbor extensive somatic hypermutation (SHM) (Pott et al., 2019) SHM may alter consensus primer binding sites, leading to allelic dropout and false-negative results, which may partially explain the high failure rate (55%) observed in the HL cohort. Despite the SHM being reported in BL, a rearrangement was not detected in only one tumor (9.1%). An hybrid-capture NGS could provide higher sensitivity in SHM, improving the sensitivity of the method (Samorodnitsky et al., 2015). Additionally, biological differences between pediatric and adult lymphomas, including distinct maturation stages and immunogenetic features, may also influence the capture efficiency of consensus primer sets (Berget et al., 2011). Also the mutation profile of BL is different according to age that indicates possible sub-group-specific stratification for understanding of biology and future treatment options (Burkhardt et al., 2022).

DLBCL, Mantle Cell Lymphoma (all more frequent in adults) and HL seem to have higher amounts of cfDNA compared to other subtypes (Ruas et al., 2023; Choi and Quintanilla-Martinez, 2025). The amount of cfDNA at diagnosis varied considerably but the small number of samples prevented the comparison of the cfDNA yield in relation to lymphoma subtypes. Even so, we found that higher cfDNA concentrations were associated with occurrence of relapse, similar to what has been reported in adult patients with DLBCL, in which cfDNA levels were associated with recurrence and tumor aggressiveness (Diez-Feijóo et al., 2024). In contrast, a study on pediatric HL (n = 155) by the AIEOP (Associazione Italiana di Emato-Oncologia Pediatrica) group found no association between cfDNA and the patients’ presentation or outcome (Primerano et al., 2016). At least one of the rearrangements identified in the tumor sample was also detected in diagnostic plasma. Some rearrangements that were poorly represented in the diagnostic cfDNA could be due to methodology limitation (Brüggemann et al., 2019; van Bladel et al., 2022; Stewart et al., 2019).

Overall, most patients showed a marked decrease in Ig/TCR ctDNA after beginning of treatment (patients #2, #14, #16, and #20) often reaching undetectable levels during remission (patients #5, #6, #8, #22, and #23). In cases of relapse, ctDNA increased again before or at the time when clinical recurrence was documented, demonstrating a concordance between the molecular and clinical course of the disease. In 17/23 cases we had more than one Ig/TCR rearrangement as a potential biomarker. With few exceptions, in tumors carrying multiple Ig/TCR rearrangements, the rearrangements evolved proportionally over the course of treatment. Differences to this trend may suggest differential clonal evolution or response to treatment, as shown in acute lymphoblastic leukemia (Szczepański et al., 2002).

Most samples contained around 2 ng of cfDNA, corresponding to 606 haploid genomes. This limited amount of cfDNA may impact the sensitivity of the assay. Therefore, a detection curve was established to determine the LOD, which was subsequently used as a reference threshold throughout sample analysis.

The small number of cases is a limitation for broader conclusions, especially considering lymphoma subgroups and genetic heterogeneity as well as the number of follow-up samples. However, monitoring ctDNA rearrangements seemed a sensitive approach to track disease burden over time. In one patient, ctDNA levels anticipated clinical relapse 8 weeks before it became evident on imaging, highlighting the potential of this technique for early detection of recurrence, although the method also showed limitations, as it failed to detect a later relapse in the same patient. Clonal evolution, oligoclonality, and recombination events may lead to instability or loss of the original rearrangements during patient’s treatment and/or disease progression, potentially resulting in false-negative MRD and limiting long-term clinical applicability (Manel et al., 2003). A dynamic “clonal tiding” in peripheral T-cell lymphomas was characterized by expansion and contraction of distinct TCR subclones during disease evolution, suggesting that TCR rearrangements may not represent stable molecular markers throughout the clinical course (Willscher et al., 2025). There is also the technical limitation in which the BIOMED-2 primers also could lose primer binding sites following SHM, which will lead to false-negative results (Pott et al., 2019).

The CAPP-Seq (Cancer Personalized Profiling by Deep Sequencing) and PhasED-seq (Phased Variant Enrichment and Detection Sequencing) both rely on the detection of tumor-specific somatic mutations and have demonstrated superior analytical sensitivity compared with conventional Ig/TCR rearrangement tracking, particularly for MRD detection in aggressive B-cell lymphomas, although their application in pediatric lymphomas remains challenging (Fu et al., 2025; Zhang et al., 2024). However, pediatric lymphomas generally harbor a lower mutational burden compared with adult lymphoid malignancies, limiting the number of trackable somatic variants available for these assays. This reduced genomic complexity may compromise the sensitivity and applicability of mutation-based ctDNA strategies in pediatric settings, particularly in tumors lacking recurrent hotspot mutations or broad genomic instability. In this context, Ig and TCR rearrangements remain highly attractive biomarkers in pediatric lymphomas, as they are tumor-specific, universally present in lymphoid malignancies, and independent of mutational burden, supporting their continued relevance for MRD monitoring in this population (Cairo et al., 2003; Burkhardt et al., 2011; Gröbner et al., 2018). Another possibility, albeit less common than Ig and TCR rearrangements, is the use of copy number alterations as molecular markers, as previously demonstrated in other pediatric solid tumors (Ruas et al., 2023).

The use of Ig and TCR rearrangements for monitoring clinical response in cfDNA in adult DLBCL was reported to improve conventional surveillance imaging with superior specificity (Roschewski et al., 2022; Kurtz et al., 2015; Scheijen et al., 2019). This study shows that the Ig and TCR are frequent in pediatric lymphoma and could be used as a biomarker in liquid biopsy. The use of ctDNA in pediatric patients with lymphoma was feasible to monitor the response to treatment and disease progression as a non-invasive and dynamic test. Therefore, due to the limited number of samples, we suggest that prospective studies with larger amounts of samples and a broader representation of lymphoma types should be performed to validate the methodology.

## Data Availability

The original contributions presented in the study are publicly available. This data can be found at NCBI BioProject with the accession number PRJNA1474705.

## Supplementary material

SUPPLEMENTARY FIGURE S1 Limit of detection (LOD) curve for IgH rearrangement detection in cfDNA. A positive sample of cfDNA containing an IgH rearrangement was serially diluted in negative cfDNA starting from 2 ng in a 1:1 dilution series, resulting in 11 dilution points. Two independent replicates were analyzed. The IgH rearrangement was consistently detected up to dilution point 7, corresponding to 0.03125 ng of cfDNA, while detection varied in the subsequent dilutions. Therefore, 0.03125 ng was defined as the minimum reliable amount of cfDNA for downstream experiments. Linear regression curves and corresponding *R* ^2^ values for each replicate are shown.

SUPPLEMENTARY FIGURE S2 Identification of rearrangements in pediatric lymphoma tumor samples. **(A)** Number of rearrangements identified by lymphoma subtype, where blue shows the number of samples with rearrangements and green shows the number of samples without rearrangements, adding the two together gives the total number of samples per subtype. **(B)** Type of rearrangement identified in each sample, where blue represents the TRD rearrangement, light green the TRG rearrangement, dark green IgL, orange IgK and red IgH. Considering the groups, the analysis of rearrangements identified the most frequent markers in different subtypes of lymphoma in **(C)** Hodgkin lymphoma, **(D)** Non-Hodgkin lymphoma and **(E)** B-cell lymphomas. Within B lymphomas we consider our largest population in **(F)** Burkitt Lymphoma.

SUPPLEMENTARY FIGURE S3 Clinical aspects of pediatric patients relevant to study. **(A)** Patients’ vital status at the time of manuscript completion. **(B)** Lymphoma classification by the Ann Arbor Staging System. **(C)** Age group of patients at diagnosis. **(D)** Sample status categorized as metastasis. **(E)** Sample status categorized as relapse.

SUPPLEMENTARY FIGURE S4 The levels of cfDNA at diagnosis. **(A,B)** cfDNA concentration (ng/mL plasma) according to tumor stage in Hodgkin lymphoma (HL) (Mann-Whitney U-test) and non-Hodgkin lymphoma (NHL) (Kruskal-Wallis test), respectively. **(C,D)** CfDNA levels at diagnosis and **(E,F)** follow up according to metastatic status in HL and NHL (Mann-Whitney U-test). **(G,H)** CfDNA levels according to relapse status in HL and NHL (p = 0.0256, Mann–Whitney U-test). **(I)** Comparison of cfDNA levels between HL and NHL cases (n = 19). P < 0.05 is indicated by *.

SUPPLEMENTARY FIGURE S5 Alluvial plot showing genetic rearrangements of Ig and TCR detected in tumor DNA (gDNA) and circulating tumor DNA (ctDNA) in the diagnosis and follow-up of patients with lymphoma. Hodgkin lymphoma (HL), nodular sclerosis (NSHL), nodular lymphocyte-predominant (NLPHL), burkitt lymphoma (BL), lymphoblastic lymphoma (LBL-B), diffuse large B-cell lymphomas (DLBCL), anaplastic large cell lymphoma (ALCL) and lymphoblastic lymphoma (LBL-T).

SUPPLEMENTARY FIGURE S6 Monitoring of patients through cfDNA (copies/mL of plasma) using Ig and TR rearrangements. The x-axis shows sampling dates and clinical stages, and the y-axis represents ctDNA copies/mL of plasma for clonal Ig/TCR rearrangements detected in plasma. Each panel corresponds to one patient. In (***)** indicates the timing of imaging evaluations on the x-axis, whereas (**−)** indicates that imaging evaluation was not performed.

SUPPLEMENTARY TABLE S1 Treatment regimens and timing of follow-up sample collection during therapy. The table summarizes treatment regimens and the timing of follow-up sample collection in relation to treatment cycles and days. Cases with unavailable information are indicated by blank. Complete Response (CR), Partial Response (PR), Stable Disease (SD), Progressive Disease (PD).

## Glossary

AIEOPj: Associazione Italiana di Emato-Oncologia Pediatrica
ALCL: Anaplastic large cell lymphoma
ALL: Acute lymphoblastic leukemia
Ampure XP: Agencourt Ampure XP beads
BL: Burkitt lymphoma
CAEE: Certificado de Apresentação para Apreciação Ética
CAPP-Seq: Cancer Personalized Profiling by Deep Sequencing
cfDNA: Cell-free DNA
CT: Computed tomography
ctDNA: Circulating tumor DNA
DLBCL: Diffuse large B-cell lymphoma
DNA: Deoxyribonucleic acid
EB: Elution buffer
EDTA: Ethylenediaminetetraacetic acid
FASTQ: Fastq format sequencing file
FR2: Framework region 2
FR3: Framework region 3
gDNA: Genomic DNA
HL: Hodgkin lymphoma
Ig: Immunoglobulin
IgH: Immunoglobulin heavy chain
IgK: Immunoglobulin kappa light chain
IgL: Immunoglobulin lambda light chain
LBL-B: B-cell lymphoblastic lymphoma
LBL-T: T-cell lymphoblastic lymphoma
LOD: Limit of Detection
MRD: Minimal residual disease
NGS: Next-generation sequencing
NHL: Non-Hodgkin lymphoma
NLPHL: Nodular lymphocyte-predominant Hodgkin lymphoma
NSHL: Nodular sclerosis Hodgkin lymphoma
PCR: Polymerase chain reaction
PET/CT: Positron emission tomography/computed tomography
PhasED-seq: Phased Variant Enrichment and Detection Sequencing
SHM: Somatic Hypermutation
TCR: T-cell receptor
TRD: T-cell receptor delta
TRG: T-cell receptor gamma
VIDJIL: High-Throughput Analysis of V(D)J Immune Repertoire platform
WHO: World Health Organization
