# Supplementary figures and images for: Feasibility of IG and TCR rearrangements quantification in ctDNA for monitoring clinical response in pediatric lymphomas
Source: Front Genet. 2026 Jun 25;17:1875204. doi: 10.3389/fgene.2026.1875204 (PMC13345596; doi:10.3389/fgene.2026.1875204)

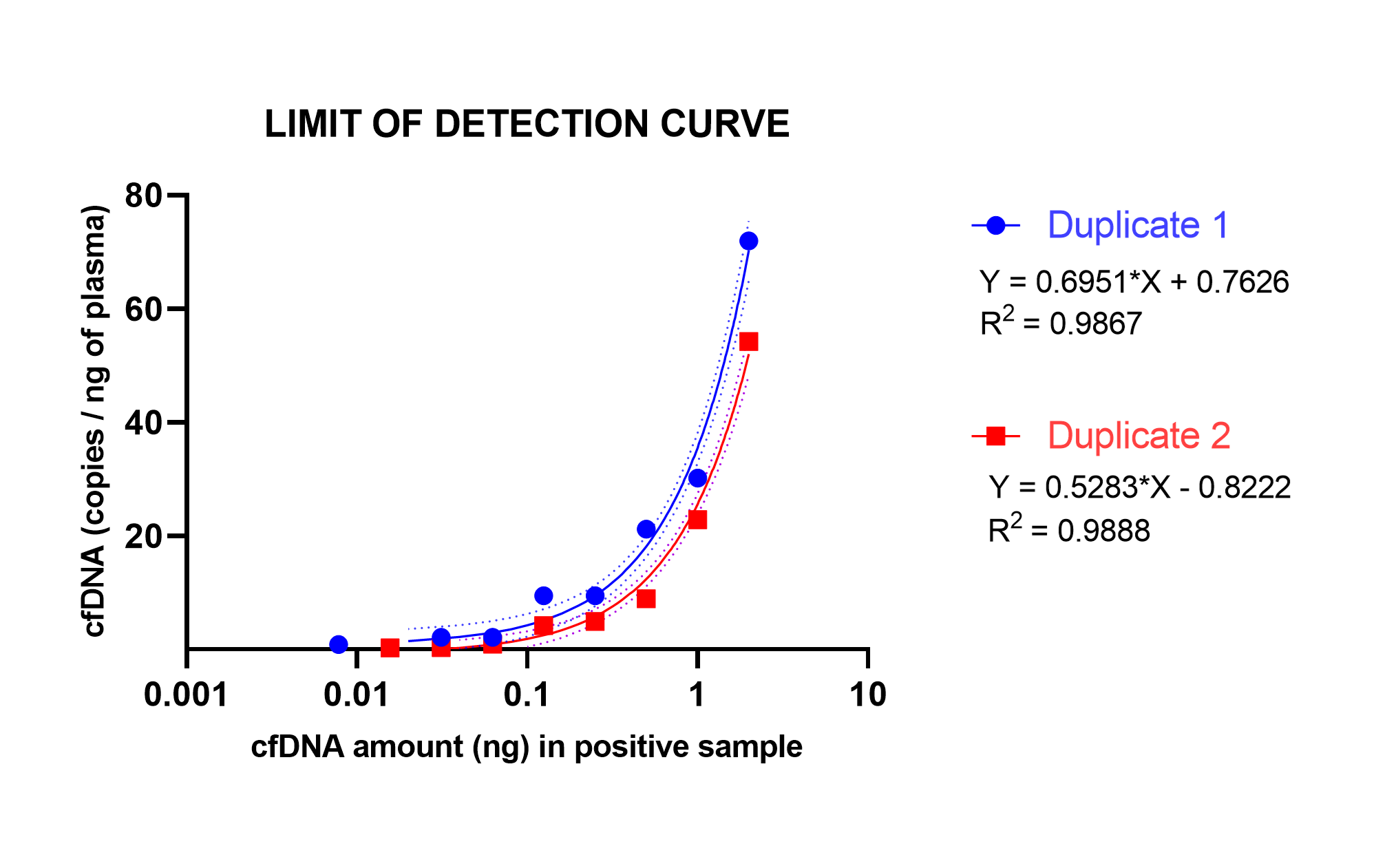

Supplement: Supplementary file 1 [file Image6.tif]

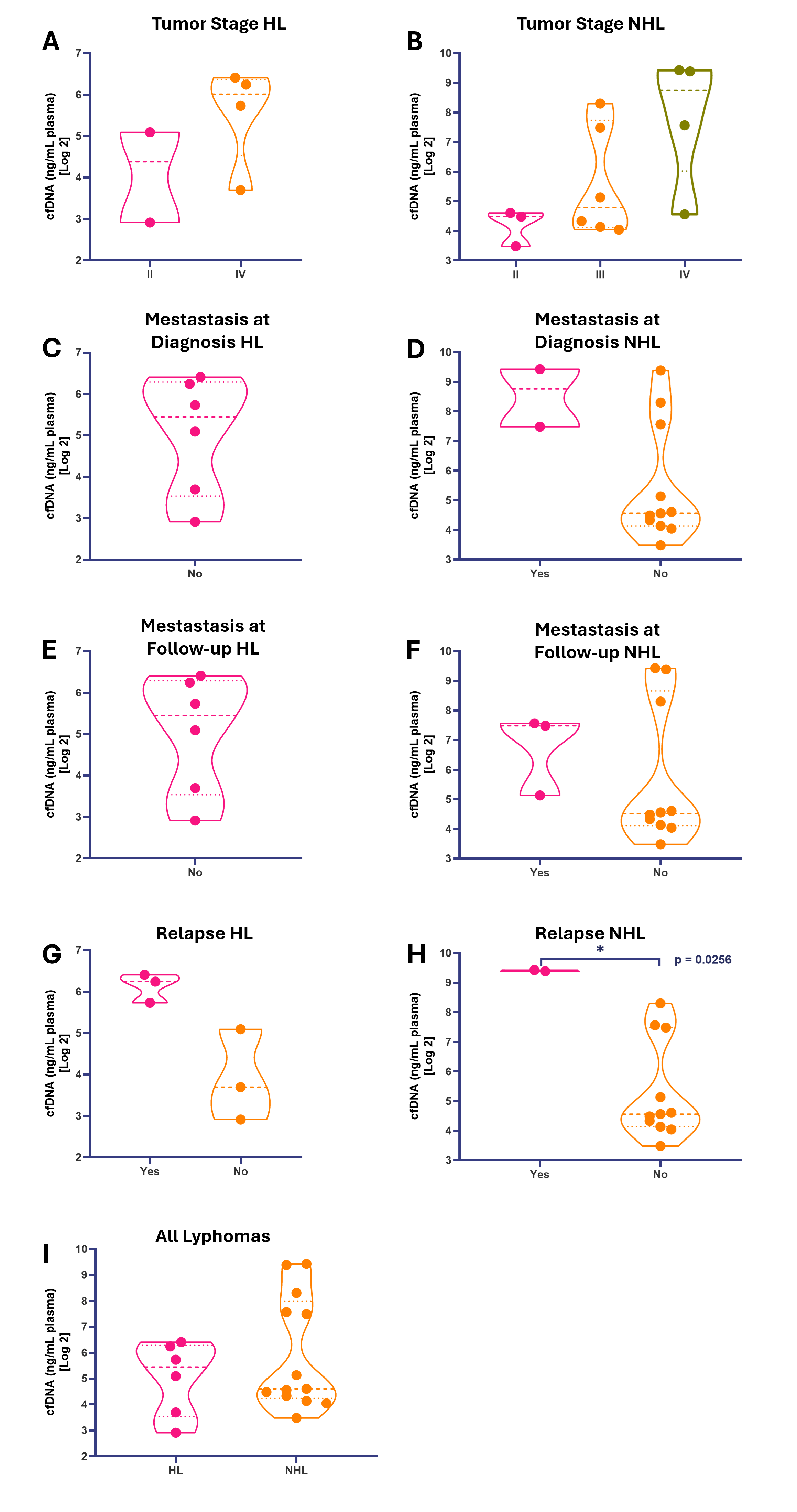

Supplement: Supplementary file 2 [file Image3.tif]

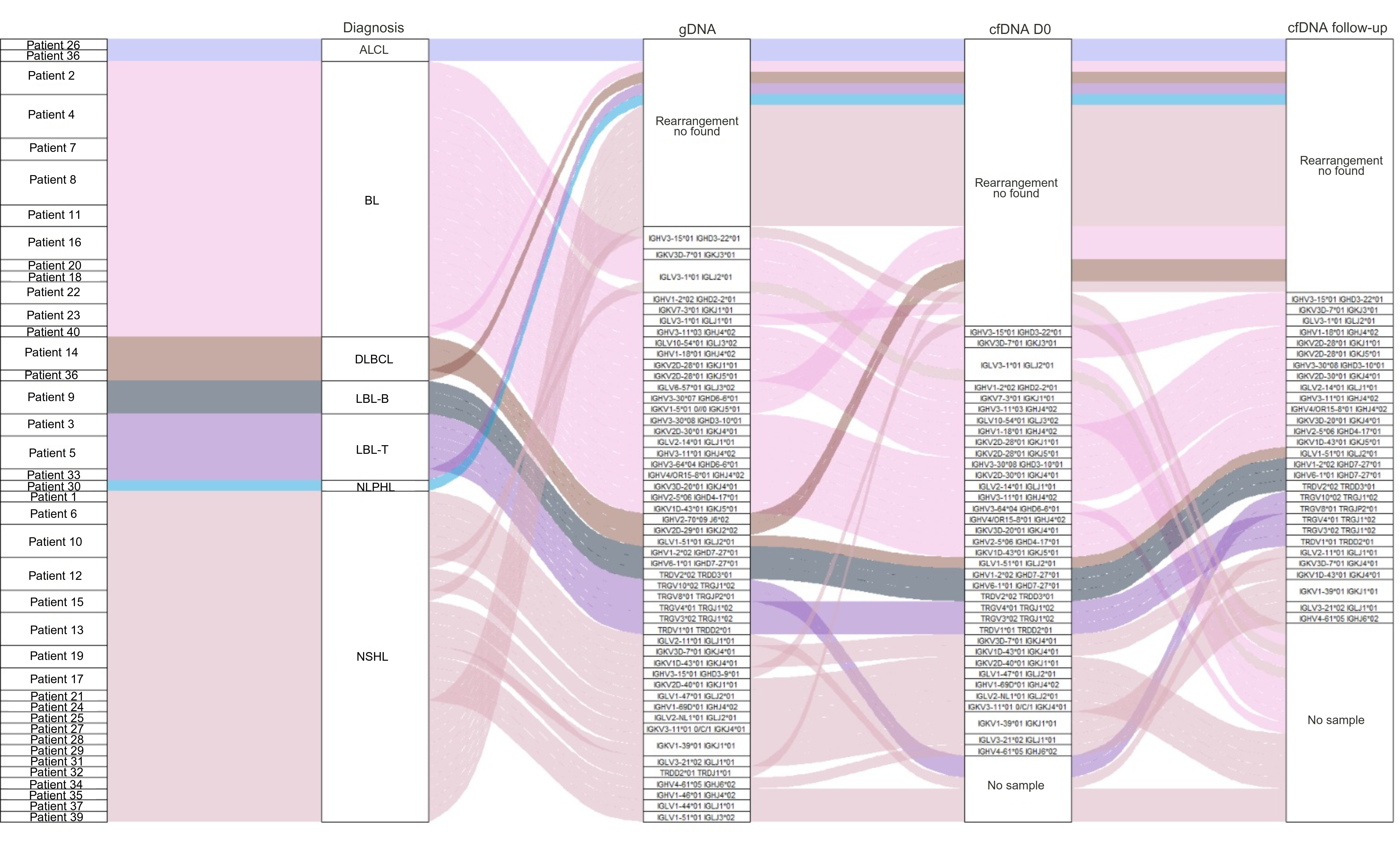

Supplement: Supplementary file 3 [file Image4.tif]

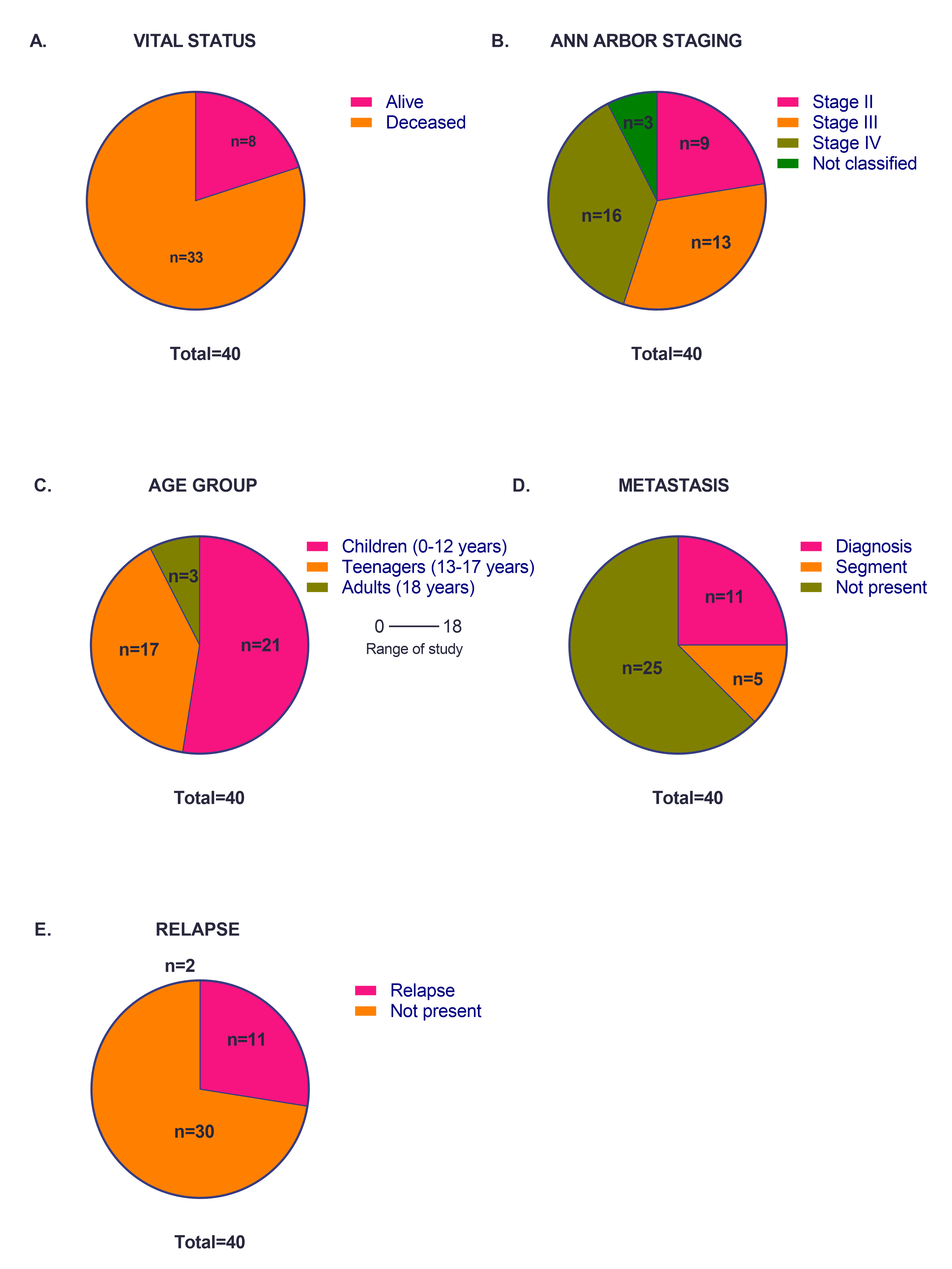

Supplement: Supplementary file 4 [file Image2.tif]

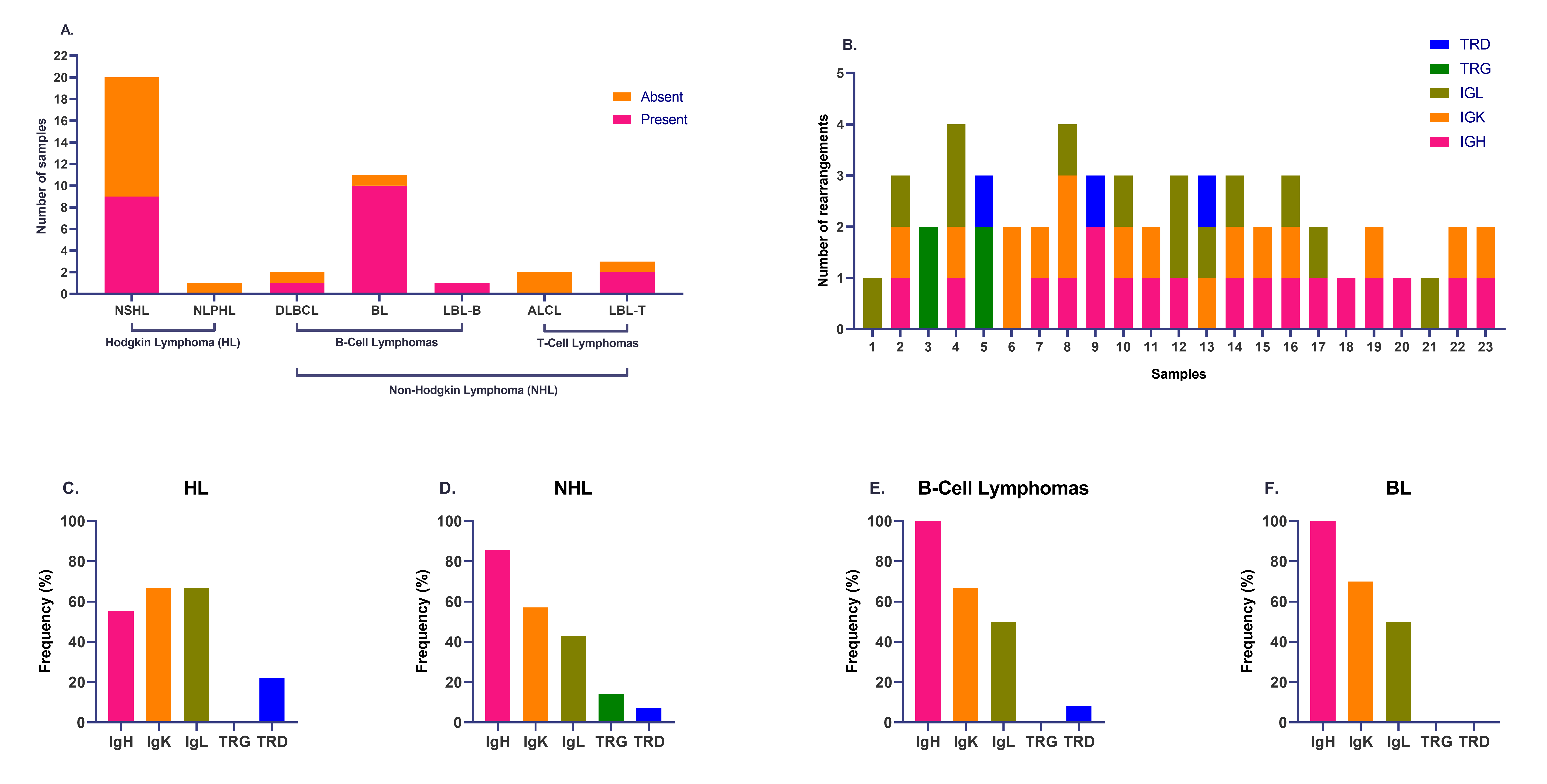

Supplement: Supplementary file 5 [file Image1.tif]

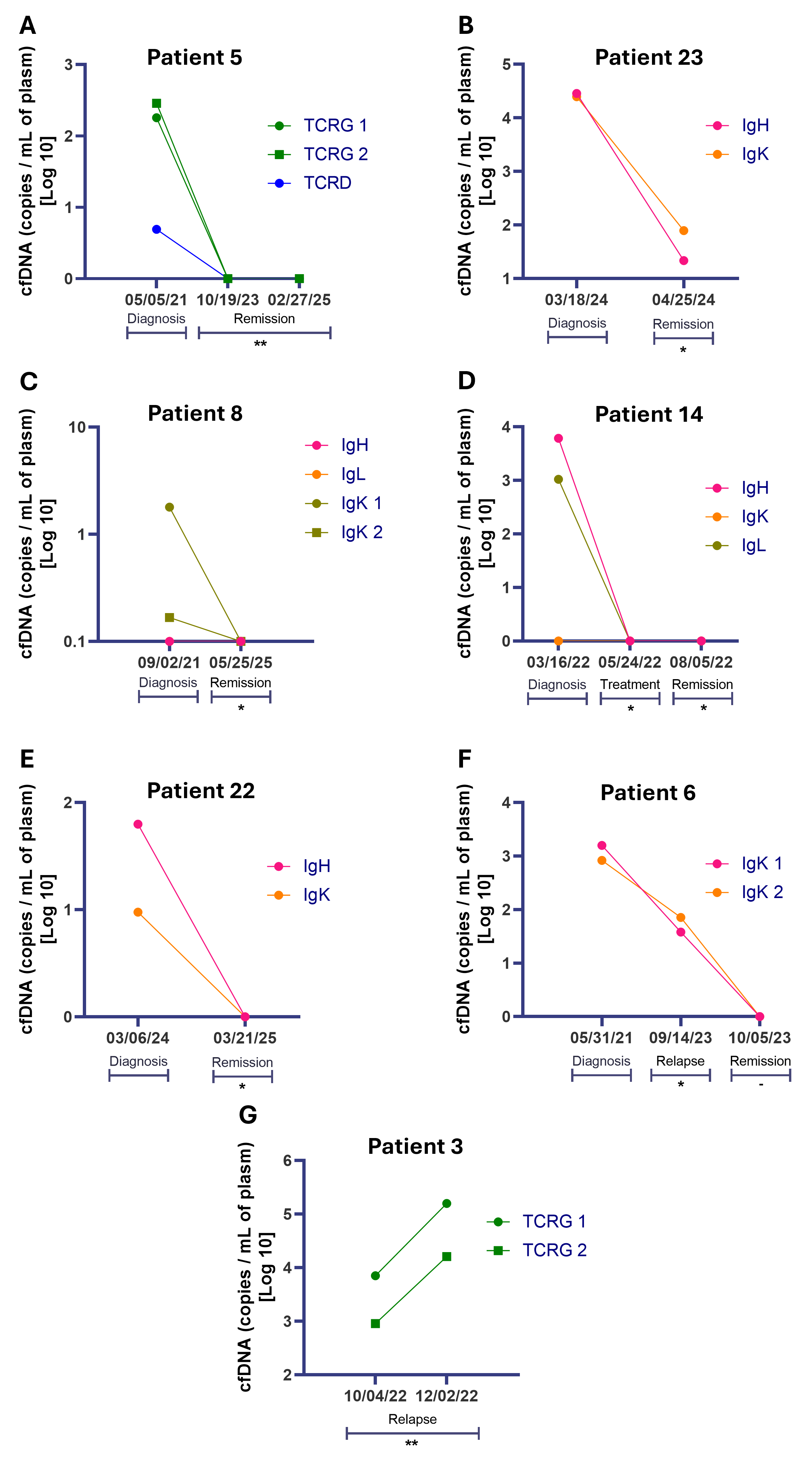

Supplement: Supplementary file 7 [file Image5.tif]
