# Supplementary material for: Feasibility of IG and TCR rearrangements quantification in ctDNA for monitoring clinical response in pediatric lymphomas
Source: Front Genet. 2026 Jun 25;17:1875204. doi: 10.3389/fgene.2026.1875204 (PMC13345596; doi:10.3389/fgene.2026.1875204)
Supplement: Supplementary file 6 [file DataSheet1.pdf]

Patients	Clinical Diagnosis	Date of Collection	Moment of Collection	Protocol	Time of Evaluation	Response /Evaluation	Method/Image
2	Burkitt Lymphoma (BL)	11/03/2020	Diagnosis	NHL-BFM 2012			
		02/01/2021	Treatment	NHL-BFM 2012	After 3º Cycle	CR	CT
		01/10/2024	Remission	NHL-BFM 2012	2 years and 9 months until the end of therapy	CR	RX thorax
3	Lymphoblastic T-Cell Lymphoma (LBL-T)	10/04/2022	Relapse	ICE	Diagnosis of relapse	PD	PET-CT + RX thorax e abdomen
		12/02/2022	Relapse	ICE	After 2º Cycle	PD	CT
5	Lymphoblastic T-Cell Lymphoma (LBL-T)	05/05/2021	Diagnosis	NHL-BFM 2012			
		10/19/2023	Remission	NHL-BFM 2012	Maintenance	CR	PET-CT
		02/27/2025	Remission	NHL-BFM 2012	21 months until the end of therapy	CR	US e RX thorax
6	Nodular Sclerosis Hodgkin Lymphoma (NSHL)	05/31/2021	Diagnosis	GPOH-HD 95			
		09/14/2023	2ª Relapse - Diagnosis	DHAP		PD	RX thorax + US
		10/05/2023	Remission	DHAP	After 1º Cycle	Not performed	Not performed
		06/03/2025	Remission	DHAP + TMO haplo + Brentuximab	After 14º Cycles of Brentux	CR	
8	Burkitt Lymphoma (BL)	09/02/2021	Diagnosis	NHL-BFM 2012			
		05/25/2022	Remission	NHL-BFM 2012	5 months until the end of therapy	CR	US e hemograma
9	Lymphoblastic B-Cell Lymphoma (LBL-B)	10/14/2121	Diagnosis	AIEOP-BFM ALL 2009			
		11/22/2021	Remission	AIEOP-BFM ALL 2009	D33	CR	DRM + PET-CT + Mielo
		02/02/2023	1ª Relapse	ALL R3	Diagnosis of relapse	PD	PET-CT + RNM
		02/03/2023	1ª Relapse	ALL R3	Diagnosis of relapse	PD	PET-CT + RNM
		09/27/2023	Treatment	R-ICE	After 4º Cycle	PR	RNM abdomen
13	Nodular Sclerosis Hodgkin Lymphoma (NSHL)	02/10/2022	Diagnosis	GPOH-HD 95			
		06/09/2022	Treatment	GPOH-HD 95	After 4º Cycle	PR	PET-CT
		08/02/2022	Treatment	GPOH-HD 95	After 6º Cycle	PR	PET-CT
		03/01/2023	1ª Relapse - Diagnosis	ICE + VNB/GEM		PD	CT
		03/03/2023	1ª Relapse - Diagnosis	ICE + VNB/GEM		PD	CT
		05/28/2024	Remission	ICE + VNB/GEM	ICE 6 cycles + VNB/GEM 6 cycles	CR	
		05/14/2025	Remission	ICE + VNB/GEM	10 months until the end of therapy	CR	US + RX thorax
14	Diffuse Large B-Cell Lymphoma (DLBCL)	03/16/2022	Diagnosis	NHL-BFM 2012			
		05/24/2022	Treatment	NHL-BFM 2012	After 2º Cycle	CR	PET-CT
		08/05/2022	Remission	NHL-BFM 2012	After 5º Cycle	CR	PET-CT
16	Burkitt Lymphoma (BL)	10/10/2022	Diagnosis	NHL-BFM 2012			
		01/03/2023	Treatment	NHL-BFM 2012	After 3º Cycle	CR	PET-CT
		02/09/2023	Treatment	NHL-BFM 2012	After 4º Cycle	Not performed	Not performed
		04/12/2023	Relapse - Diagnosis	R-ICE		PD	PET-CT + CT
19	Nodular Sclerosis Hodgkin Lymphoma (NSHL)	11/23/2022	Diagnosis	GPOH-HD 95			
		01/31/2023	Treatment	GPOH-HD 95	After 2º Cycle	CR	PET-CT
		03/29/2023	Treatment	GPOH-HD 95	After 4º Cycle	CR	US + RX thorax
		05/03/2023	1ª Relapse - Diagnosis	ICE		PD	PET-CT
		05/11/2023	1ª Relapse - Diagnosis	ICE		PD	PET-CT
		07/19/2023	Tratamento	ICE	After 2º Cycle	CR	PET-CT
		10/11/2023	2ª Relapse - Treatment	DHAP	After 2º Cycle	CR	PET-CT
		07/31/2024	Treatment	DHAP + TMO autolog + Brentuximab	After 8 Cycles of Brentux	CR	PET-CT
		03/11/2025	Remission	DHAP + TMO autolog + Brentuximab	5 months until the end of therapy	Not performed	Not performed
		04/15/2025	Remission	DHAP + TMO autolog + Brentuximab	6 months until the end of therapy	CR	PET-CT
20	Burkitt Lymphoma (BL)	12/05/2022	Diagnosis	NHL-BFM 2012			
		11/08/2023	Treatment	NHL-BFM 2012	8 months until the end of therapy	CR	US
		02/19/2025	Remission	NHL-BFM 2012	1 year and 10 months until the end of therapy	CR	US
22	Burkitt Lymphoma (BL)	03/06/2024	Diagnosis	NHL-BFM 2012			
		03/21/2025	Remission	NHL-BFM 2012	9 months until the end of therapy	CR	US
23	Burkitt Lymphoma (BL)	03/18/2024	Diagnosis	NHL-BFM 2012			
		25/04/2024	Remission	NHL-BFM 2012	After 1º Cycle	CR	CT
